# Supplementary material for: Michael Acceptor Compounds as Hemoglobin Oxygen Affinity Modulators for Reversing Sickling of Red Blood Cells
Source: Pharmaceuticals (Basel). 2025 May 24;18(6):783. doi: 10.3390/ph18060783 (PMC12196294; doi:10.3390/ph18060783)
Supplement: Supplementary file 1 [file pharmaceuticals-18-00783-s001.zip › pharmaceuticals-3624798-supplementary.pdf]

SUPPLEMENTARY MATERIAL

Michael Acceptor Compounds as Hemoglobin Oxygen Affinity Modulators for Reversing Sickling of Red Blood Cells

Khadijah A. Mohammad [1], Asala H. Naghi [1], Mohini S. Ghatge [2], Benita Balogun [3], Mariana Macias [3], Salma Roland [3], Albert Opare [2], Osheiza Abdulmalik [3], Martin K. Safo [2,*], Abdelsattar M. Omar [1] and Moustafa E. El-Araby [2,4]

[1] Department of Pharmaceutical Chemistry, Faculty of Pharmacy, King Abdulaziz University,
Jeddah 21589, Saudi Arabia; kmohammad@kau.edu.sa (K.A.M.);
asala.naghi@gmail.com (A.H.N.);
asmansour@kau.edu.sa (A.M.O.)

[2] Department of Medicinal Chemistry, School of Pharmacy and Center for Drug Discovery, Virginia
Commonwealth University, Richmond, VA 23219, USA; msghatge@vcu.edu (M.S.G.);
oparea@vcu.edu (A.O.); dawoudme@vcu.edu (M.E.E.-A.)

[3] Division of Hematology, The Children's Hospital of Philadelphia, Philadelphia, PA 19104, USA; benitab@sas.upenn.edu (B.B.); mariana7@sas.upenn.edu (M.M.); rsal@sas.upenn.edu (S.R.); abdulmalik@email.chop.edu (O.A.)

[4] Department of Pharmaceutical Organic Chemistry, Faculty of Pharmacy, Helwan University, Ain Helwan, Cairo 11795, Egypt

* Correspondence: msafo@vcu.edu; Tel.: +1-804-828-7291

Spectra of Compounds:

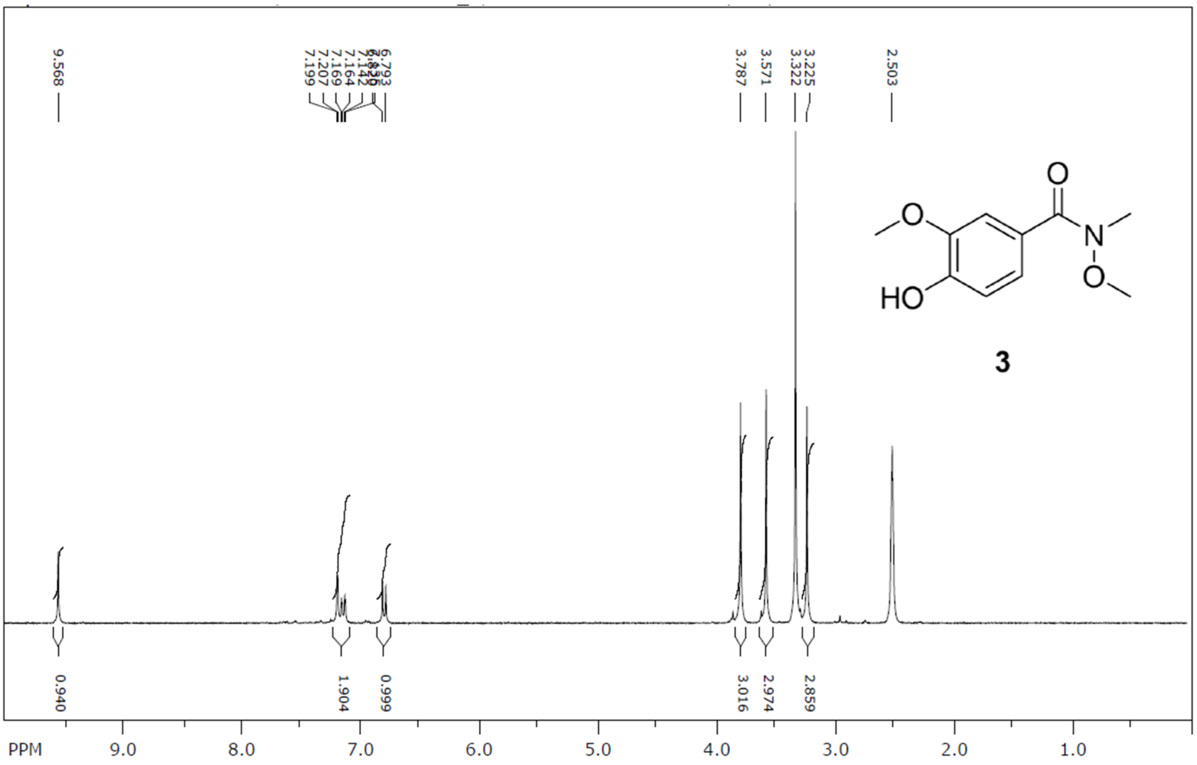

Figure S1. ^{1}H NMR spectrum of 4-hydroxy-N,3-dimethoxy-N-methylbenzamide (3) recorded in DMSO-d_6 at 300 MHz. All expected proton resonances are observed, including a singlet at δ 9.57 corresponding to the phenolic –OH proton.

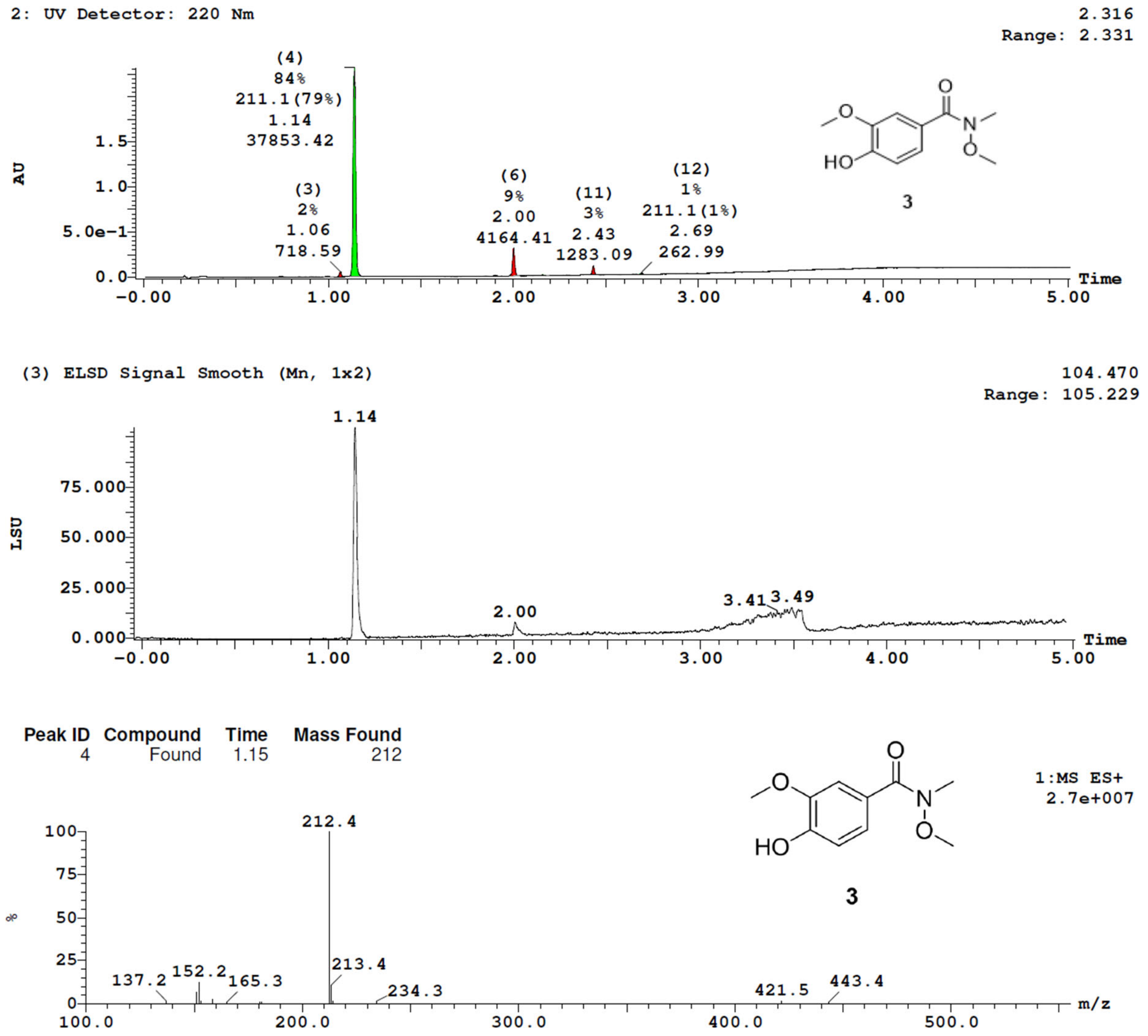

Figure S2. LC–MS chromatogram (UPLC–ESI$^+$) of 4-hydroxy-N,3-dimethoxy-N-methylbenzamide (3). A major product peak is observed (approximately 84% of total UV area, 220 nm), with an $[M+H]^+$ molecular ion at m/z 212 in the positive-ion mass spectrum.

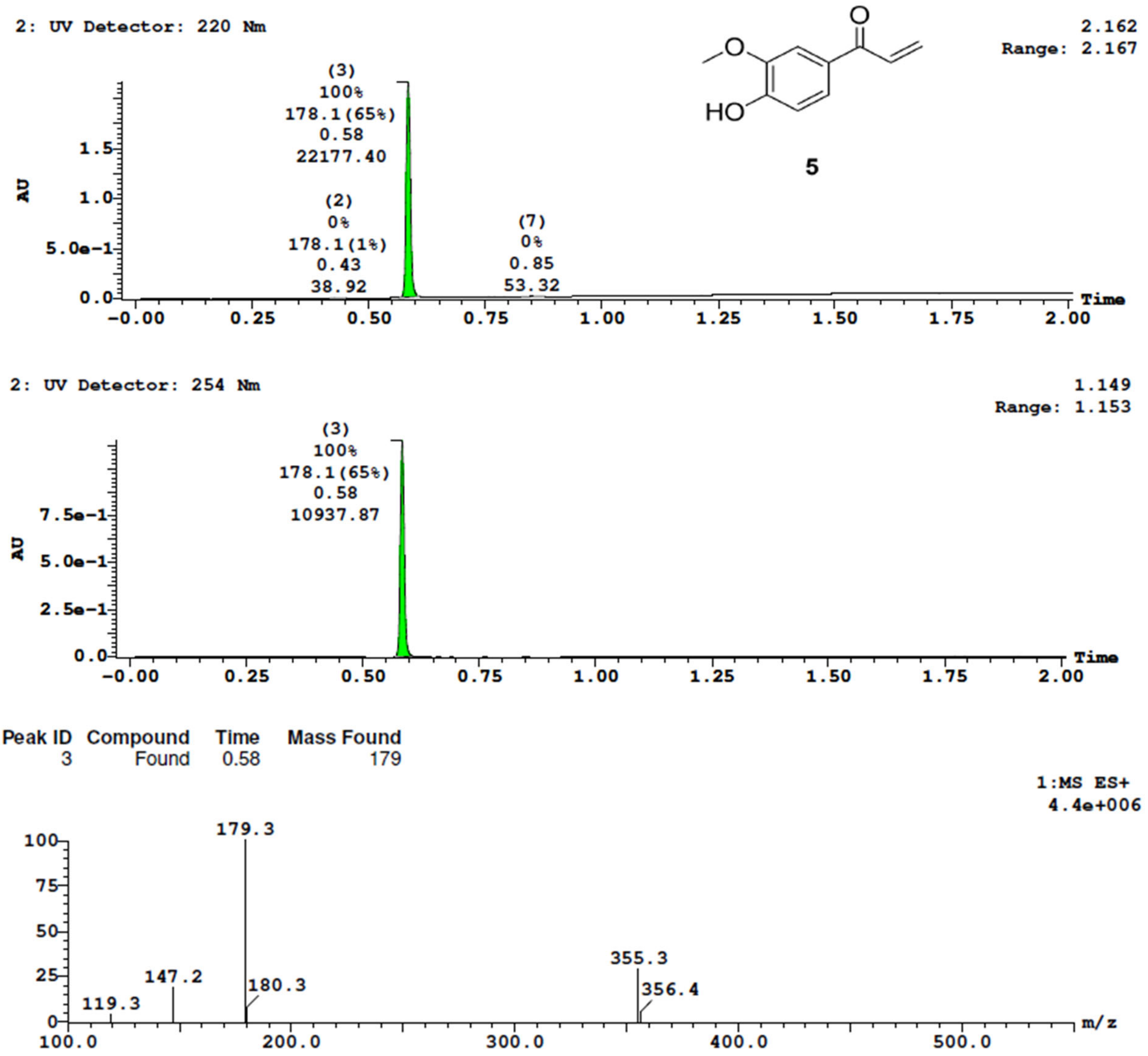

Figure S3. LC–MS analysis of 1-(4-hydroxy-3-methoxyphenyl)prop-2-en-1-one (5). The chromatogram displays a single major product peak (~99% purity by UV area), and the ESI mass spectrum shows the protonated molecular ion [M+H] + at m/z 179.

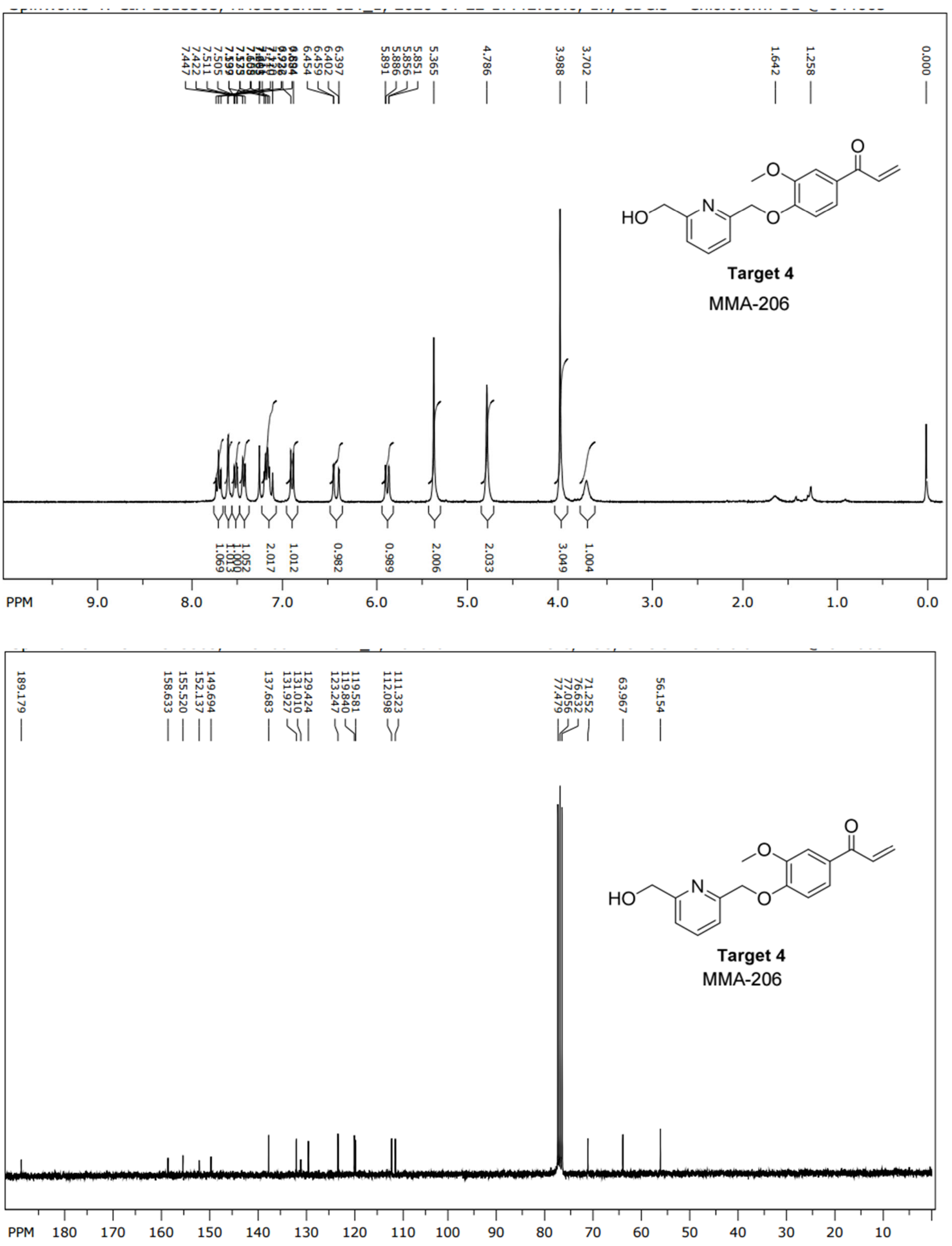

Figure S4. ^{1}H NMR spectrum of 1-(4-((6-(hydroxymethyl)pyridin-2-yl)methoxy)-3-methoxyphenyl)prop-2-en-1-one (MMA-206) in $CDCl_3$ at 300 MHz. All expected aromatic (δ 6.9–7.7) and vinylic protons are present, along with the benzyloxymethylene protons appearing as singlets at δ ~5.38 and 4.80 (CH_2–O– linking the pyridylmethyl group) and a methoxy singlet at δ 4.00.

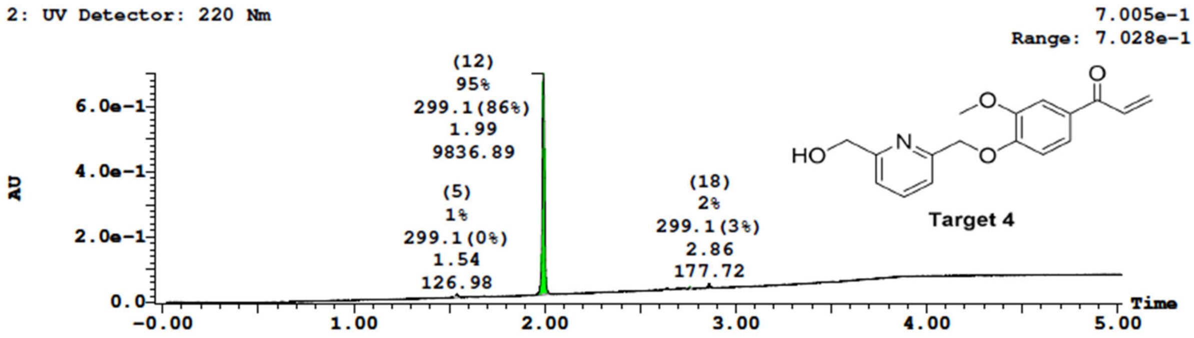

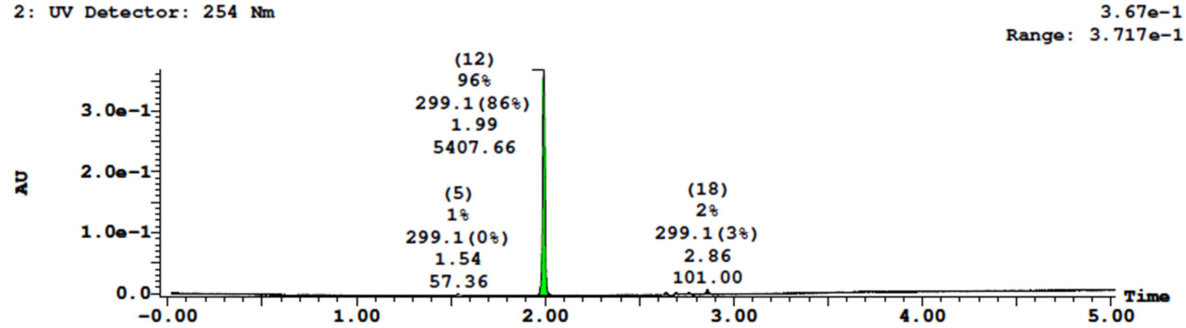

Peak ID	Compound	Time	Area %BP	Mass Found
12	Found	1.99	100	300

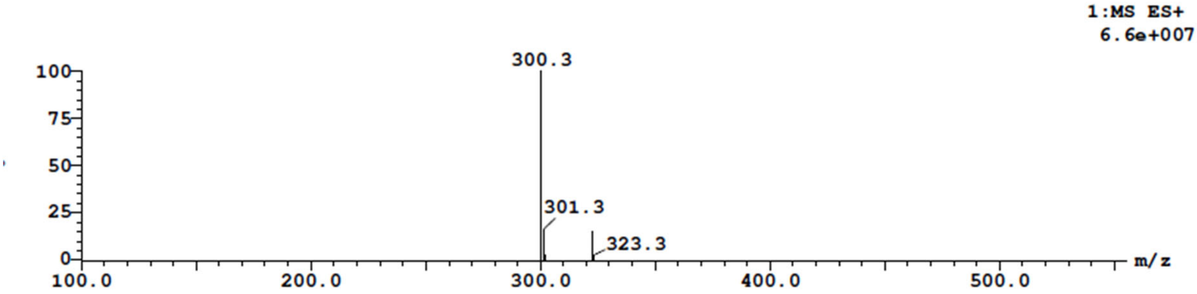

Figure S5. LC–MS chromatogram of 1-(4-((6-(hydroxymethyl)pyridin-2-yl)methoxy)-3-methoxyphenyl)prop-2-en-1-one (MMA-206). A dominant product peak is observed (~95% purity by area), and the ESI mass spectrum shows the $[M+H]^+$ ion at m/z 300, corresponding to the molecular ion.

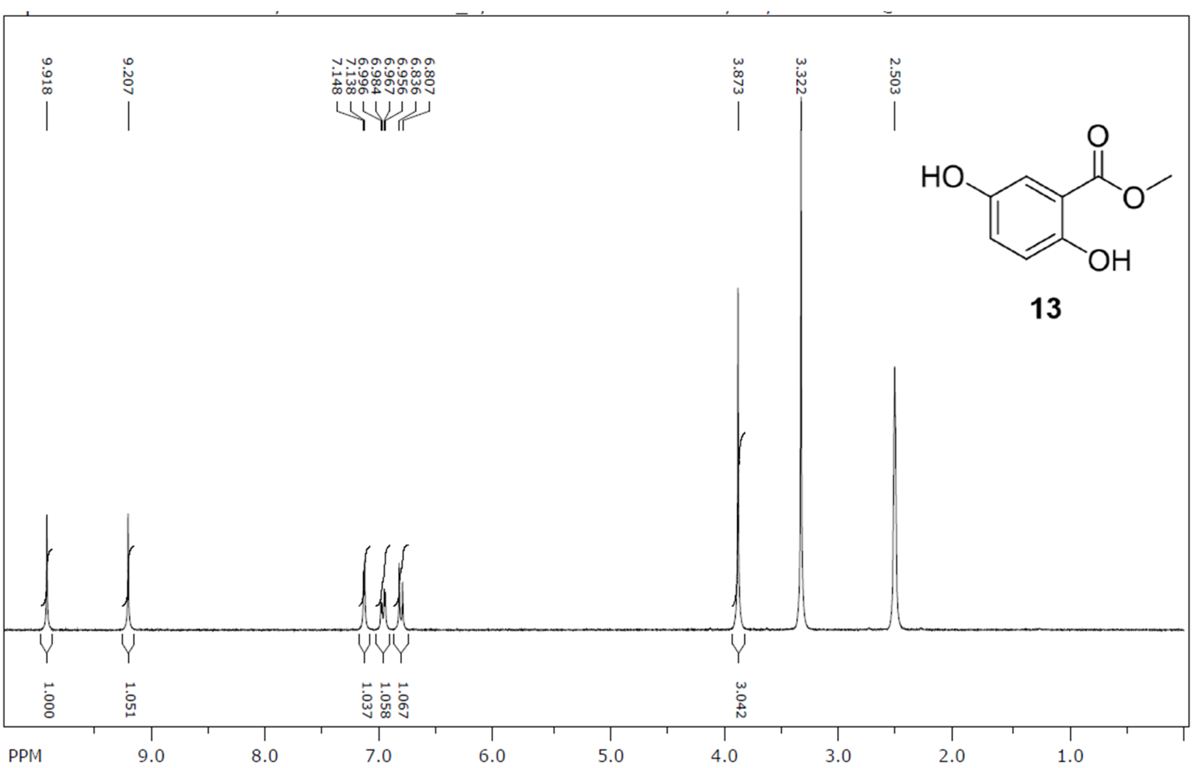

Figure S6. ^{1}H NMR spectrum of methyl 2,5-dihydroxybenzoate (13) in DMSO-d_6, 300 MHz. Two downfield singlets at δ 9.92 and 9.21 are observed for the aromatic hydroxyl protons (H-2 and H-5), along with aromatic proton signals at δ 6.82–7.14 and a methoxy singlet at δ 3.87.

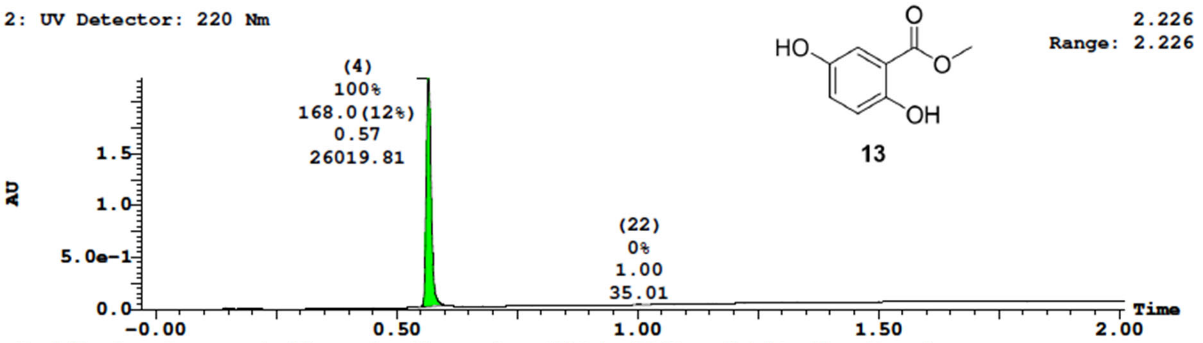

Peak Number	Compound	Time	AreaAbs	Area %Total	Width	Height	Mass Found
4	Found	0.57	3e+004	99.87	0	2e+006	168.0423
22		1.00	4e+001	0.13	0	3e+003	Not Found

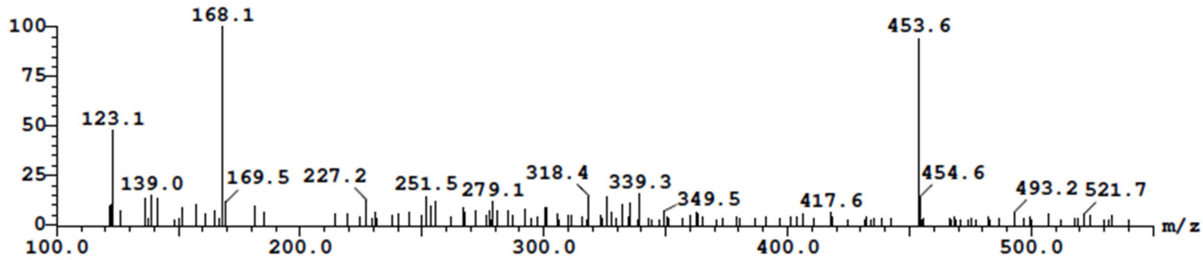

Figure S7. LC–MS analysis of methyl 2,5-dihydroxybenzoate (13). The UPLC chromatogram (220 nm) shows a single product peak (~100% purity); however, no molecular ion is detected in the positive ESI–MS (no $[M+H]^+$ observed, consistent with very poor ionization of this dihydroxybenzoate under the conditions used).

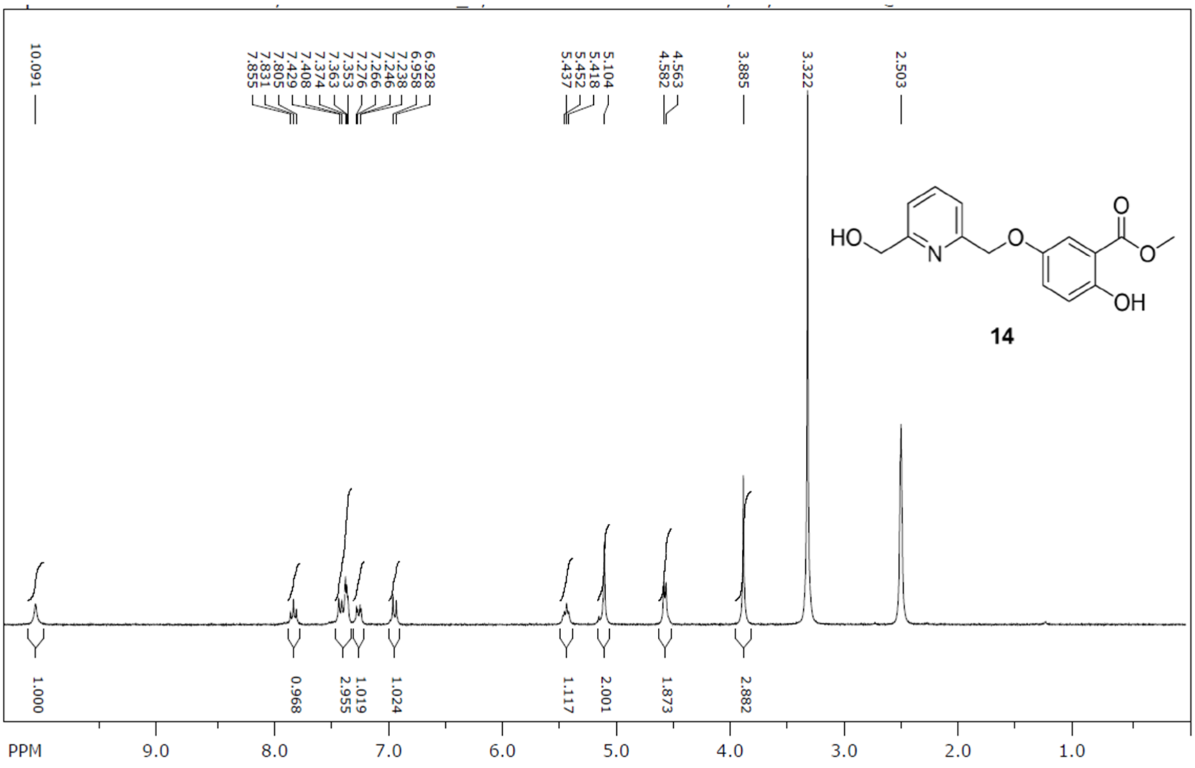

Figure S8. ^{1}H NMR spectrum of methyl 2-hydroxy-5-((6-(hydroxymethyl)pyridin-2-yl)methoxy)benzoate (14) in DMSO-d_6 at 300 MHz. A singlet at δ 10.09 is assigned to the phenolic OH (H-2). The spectrum also shows the aromatic proton multiplets (δ ~7.3–7.8), the benzyloxymethyl protons as a triplet at δ 5.44 (1H, –CH_2OH), and a doublet at δ 4.57 (2H, CH_2–OH), a benzylic OCH_2 singlet at δ 5.10 (2H, Ar–O–CH_2–), and a methyl ester singlet at δ 3.89.

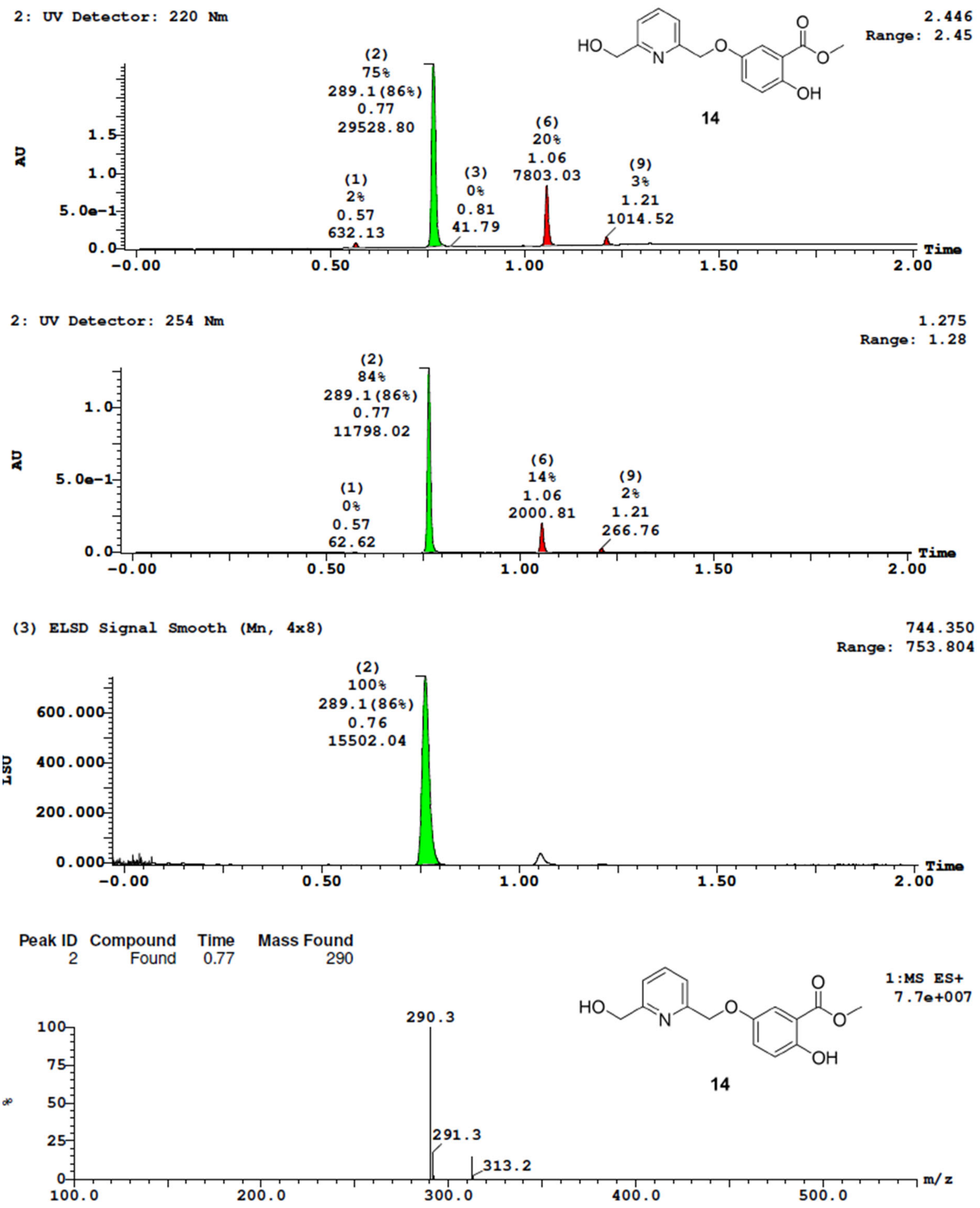

Figure S9. LC–MS chromatogram of methyl 2-hydroxy-5-((6-(hydroxymethyl)pyridin-2-yl)methoxy)benzoate (14). The positive-ion mass spectrum shows the protonated molecule $[M+H]^+$ at m/z 290. The 220 nm UV trace indicates ~75% purity for the product (a dominant product peak with some minor impurity peaks).

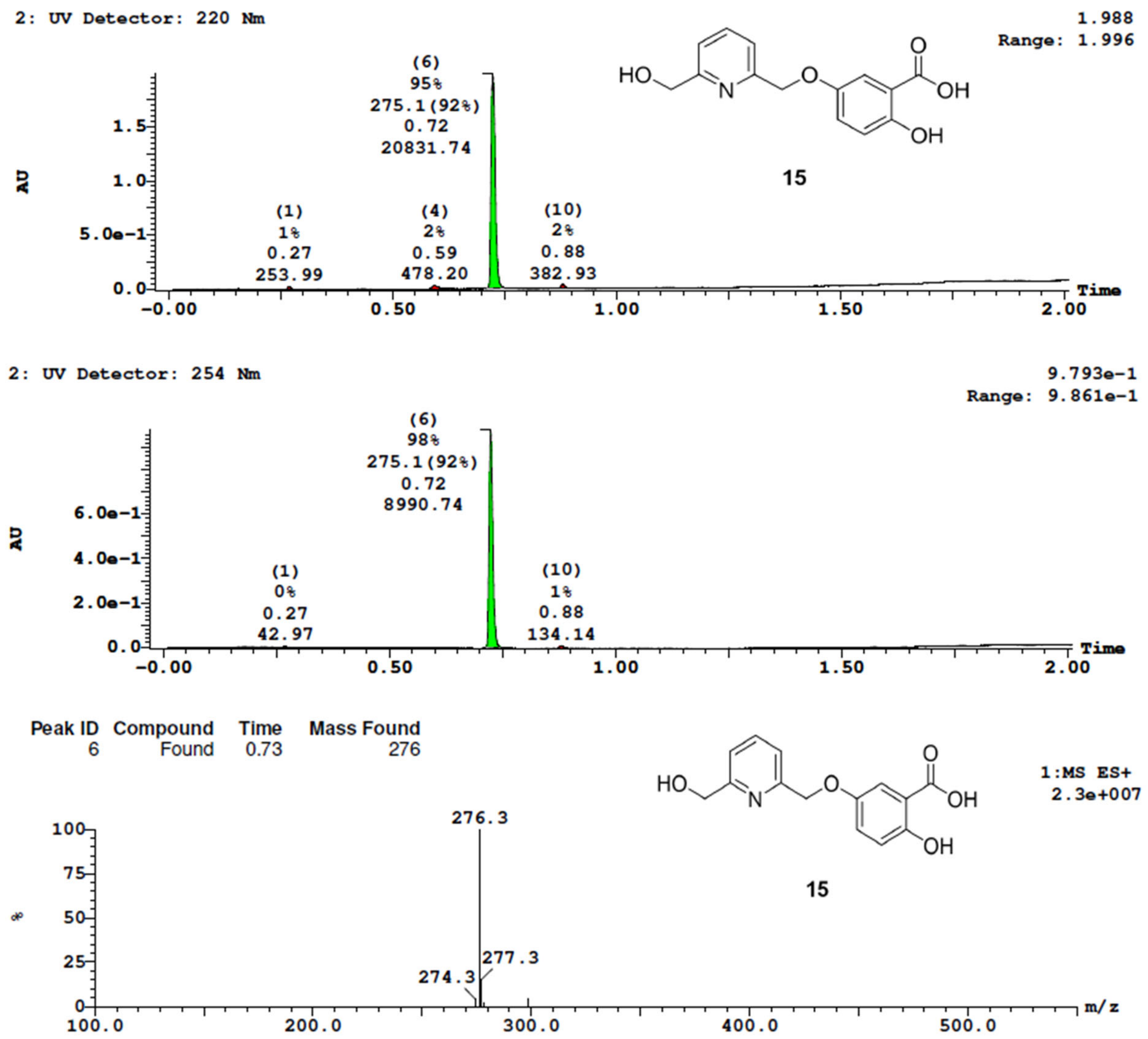

Figure S10. LC–MS of 2-hydroxy-5-((6-(hydroxymethyl)pyridin-2-yl)methoxy)benzoic acid (15). The mass spectrum shows $[M+H]^+$ at m/z 276, and the LC trace displays a single predominant peak (~95% purity by area) corresponding to the product.

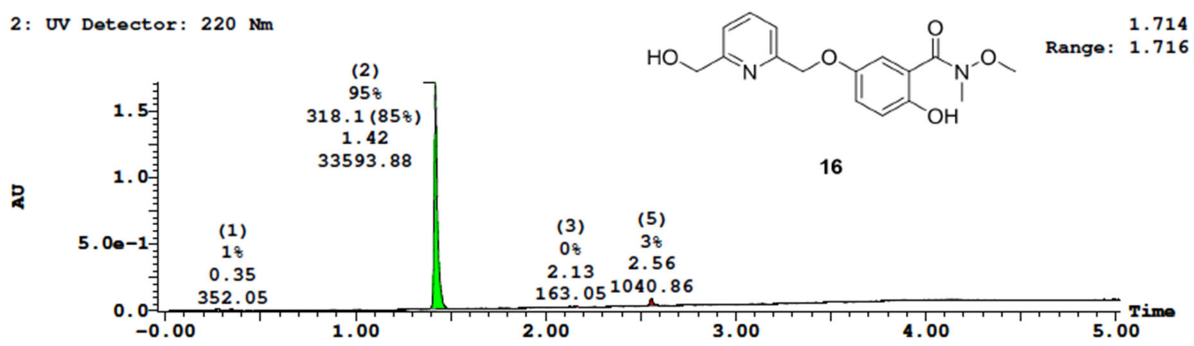

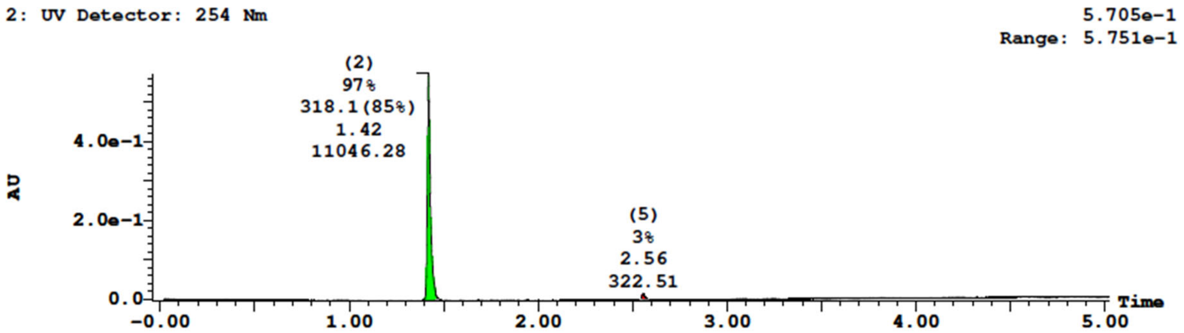

Peak Number	Compound	Time	AreaAbs	Area %Total	Width	Height	Mass Found
2	Found	1.42	1e+004	97.16	0	6e+005	318.1216
5		2.56	3e+002	2.84	0	2e+004	Not Found

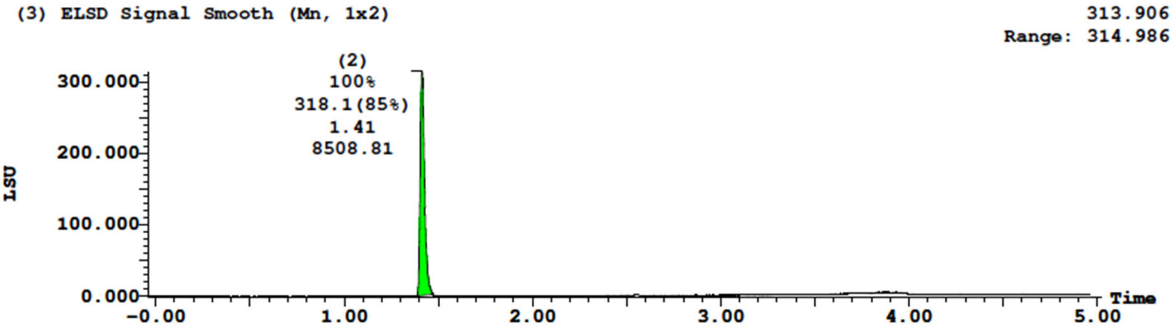

Peak ID	Compound	Time	Area %BP	Mass Found
2	Found	1.42	100	319

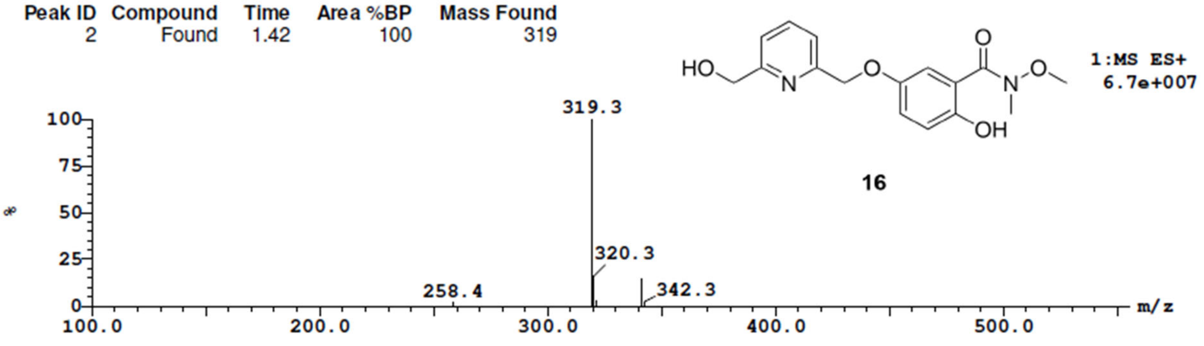

Figure S11. LC–MS analysis of intermediate 16 (2-hydroxy-5-((6-(hydroxymethyl)pyridin-2-yl)methoxy)-N-methoxy-N-methylbenzamide). The ESI mass spectrum shows the molecular ion as $[M+H]^+$ at m/z 319. The UPLC chromatogram indicates a major product peak (~95% purity) with no significant side-products.

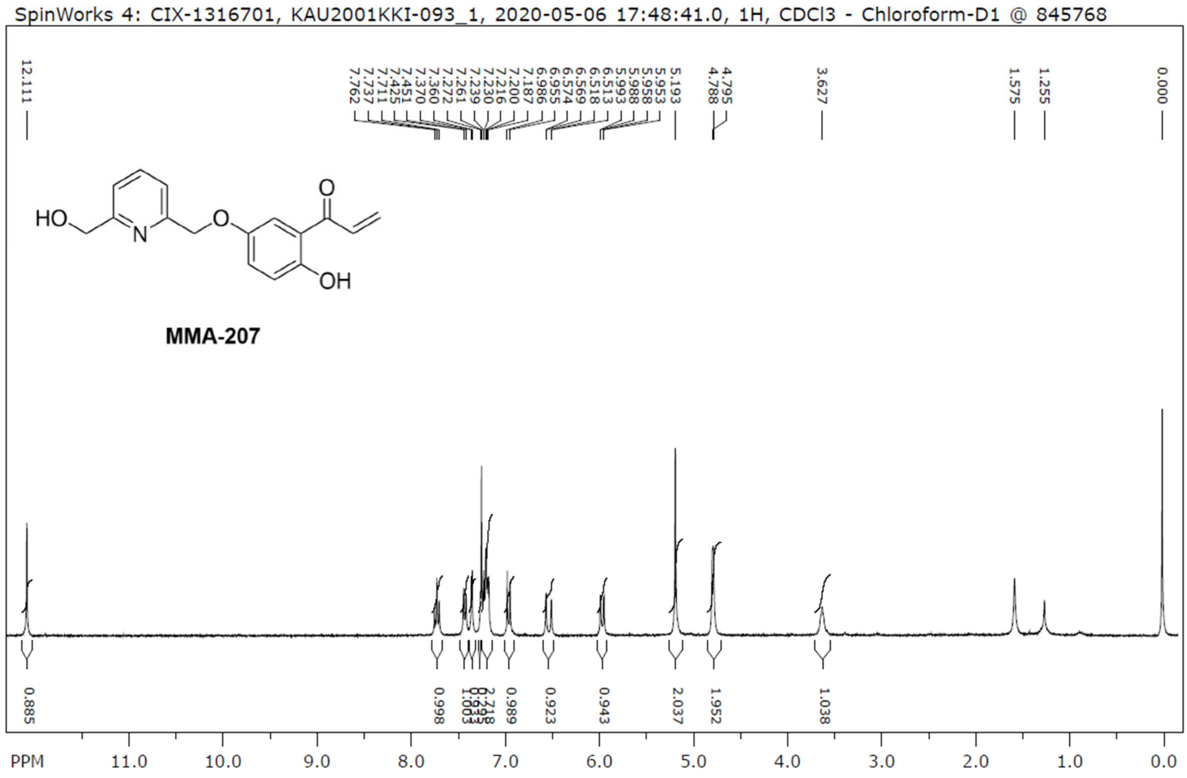

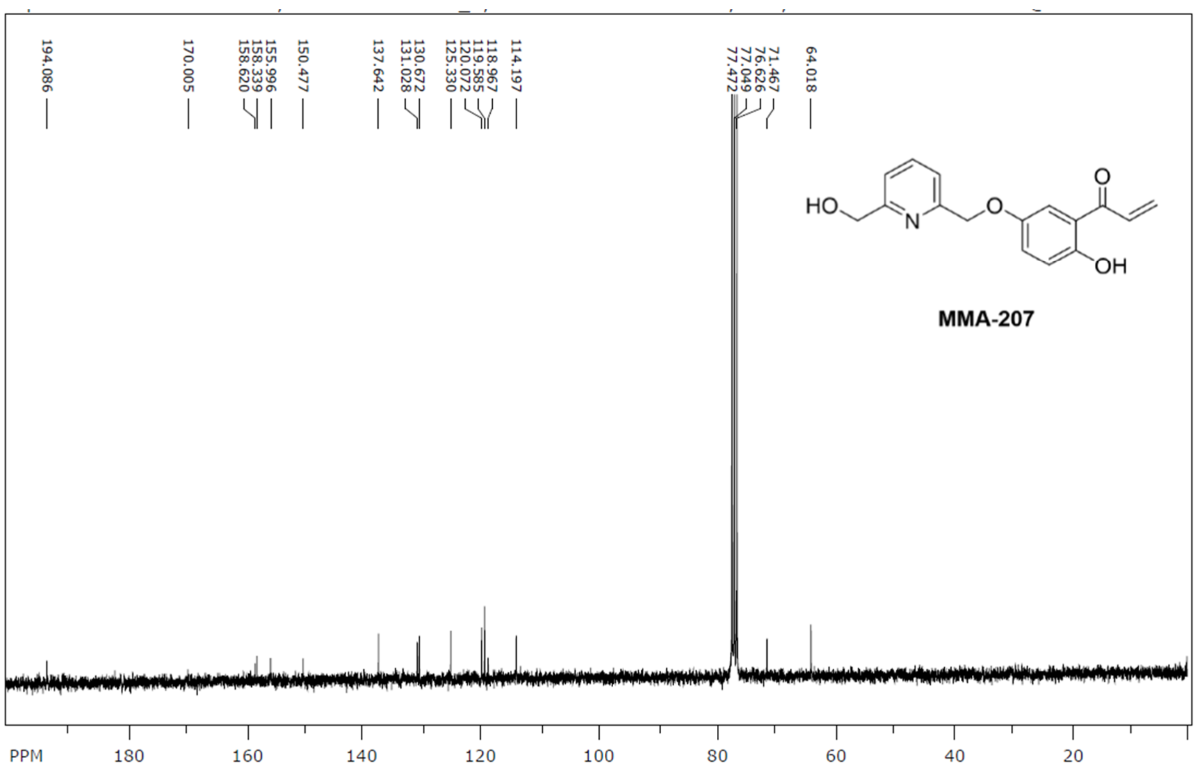

Figure S12. ^{1}H NMR spectrum of 1-(2-hydroxy-5-((6-(hydroxymethyl)pyridin-2-yl)methoxy)phenyl)prop-2-en-1-one (MMA-207) in $CDCl_3$ at 300 MHz. A very downfield singlet at δ 12.13 corresponds to the intramolecularly hydrogen-bonded phenolic proton (H-2). The aromatic and olefinic protons are observed between δ 6.56–7.76, and the benzyloxymethylene linker protons appear as singlets at δ 5.21 and 4.81 (each 2H, Ar–O–CH_2– and –CH_2OH attached to the pyridyl ring).

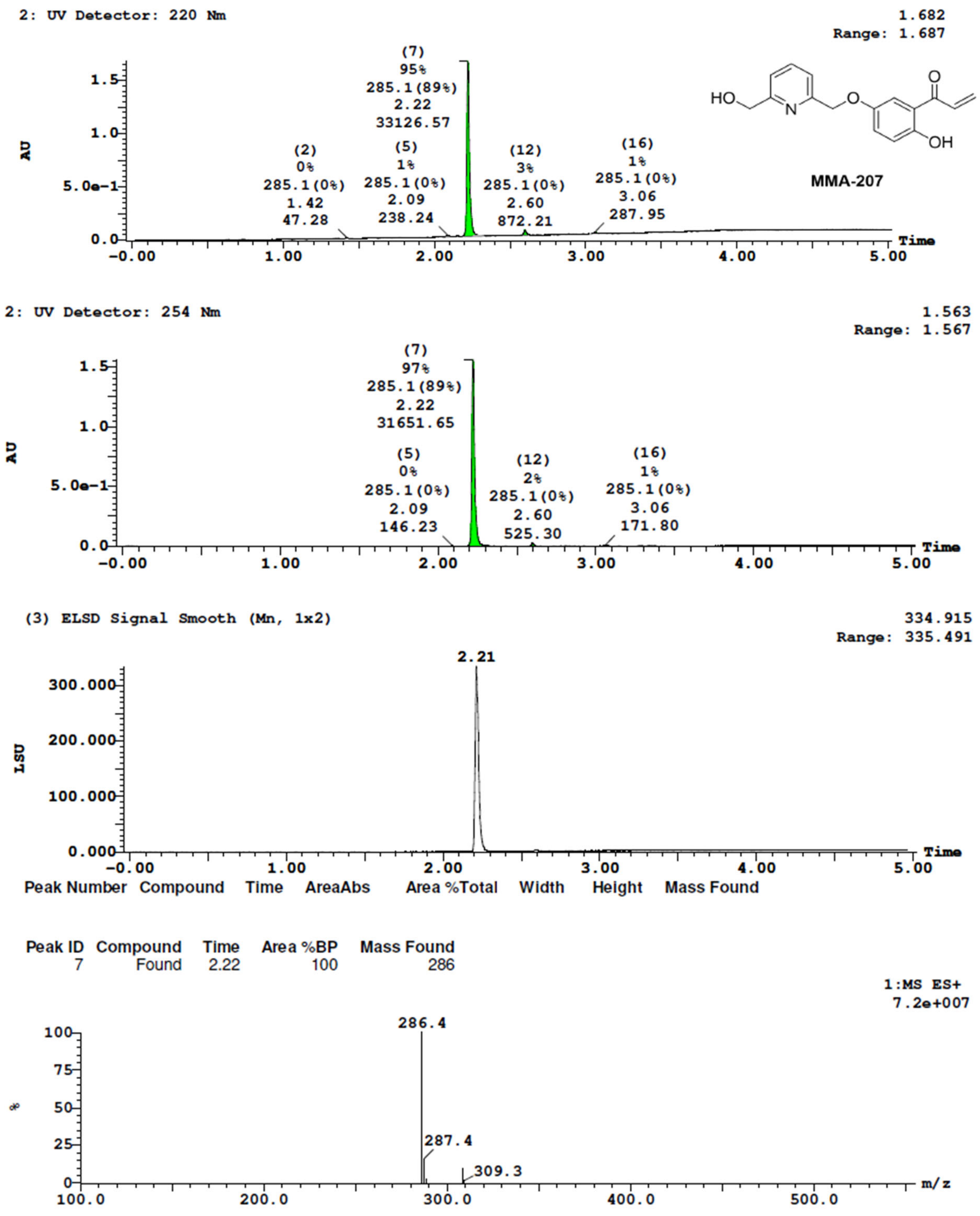

Figure S13. LC–MS of 1-(2-hydroxy-5-((6-(hydroxymethyl)pyridin-2-yl)methoxy)phenyl)prop-2-en-1-one (MMA-207). The analysis shows a major product peak (~95% purity) with an observed ion at m/z 286, corresponding to the sodium adduct $[M+Na]^+$ (no $[M+H]^+$ was detected in the positive ESI).

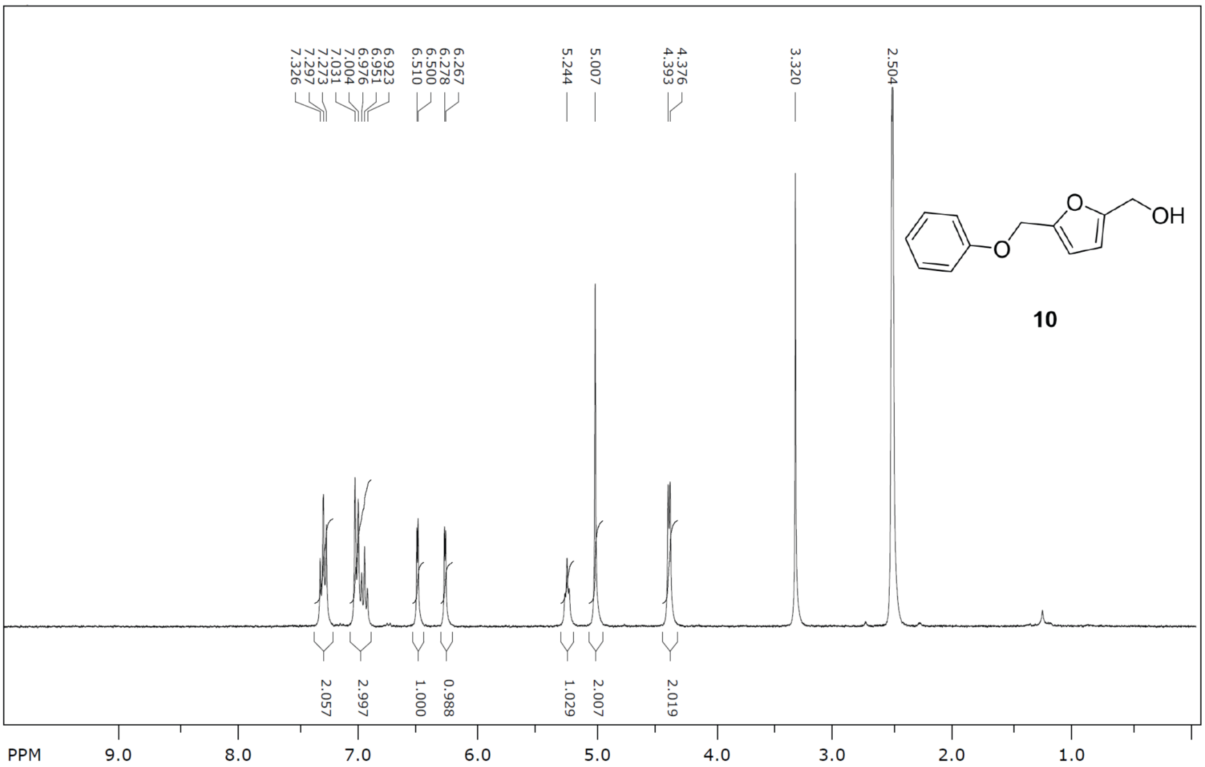

Figure S14. ^{1}H NMR spectrum of (5-(phenoxymethyl)furan-2-yl)methanol (10) in DMSO-d_6, 300 MHz. The phenoxymethyl bridge methylene protons appear as a singlet at δ 5.01 (2H, Ph–O–CH_2–furan). The furan–CH_2OH group is evident from a doublet at δ 4.38 (2H, CH_2–OH, J ≈ 5.1 Hz) and a corresponding hydroxyl triplet at δ 5.24 (1H, –OH, J ≈ 5.3 Hz). The aromatic protons of the phenyl ring and the furan ring resonate in the range δ 6.27–7.30.

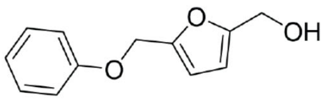

10

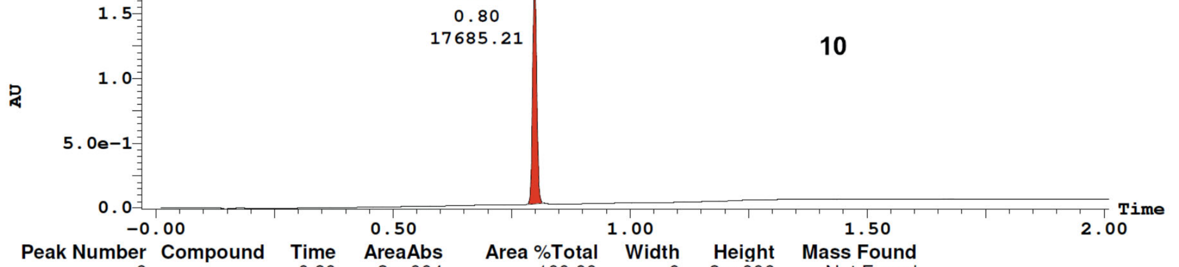

Peak Number	Compound	Time	AreaAbs	Area %Total	Width	Height	Mass Found
6		0.80	2e+004	100.00	0	2e+006	Not Found

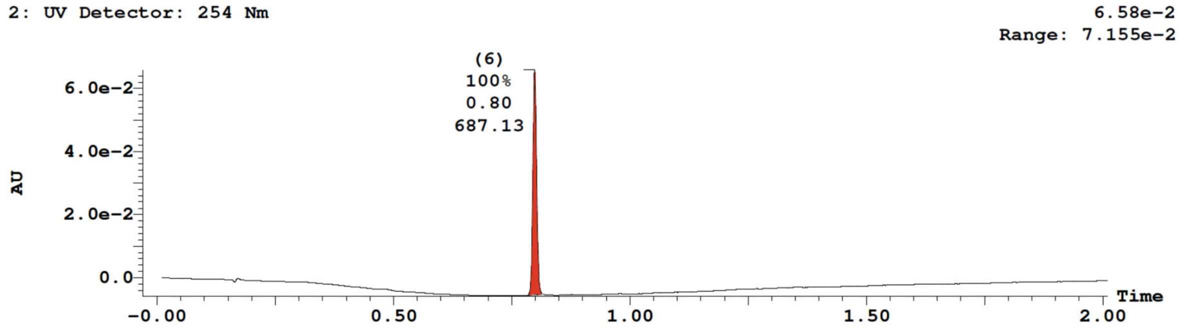

Peak Number	Compound	Time	AreaAbs	Area %Total	Width	Height	Mass Found
6		0.80	7e+002	100.00	0	7e+004	Not Found

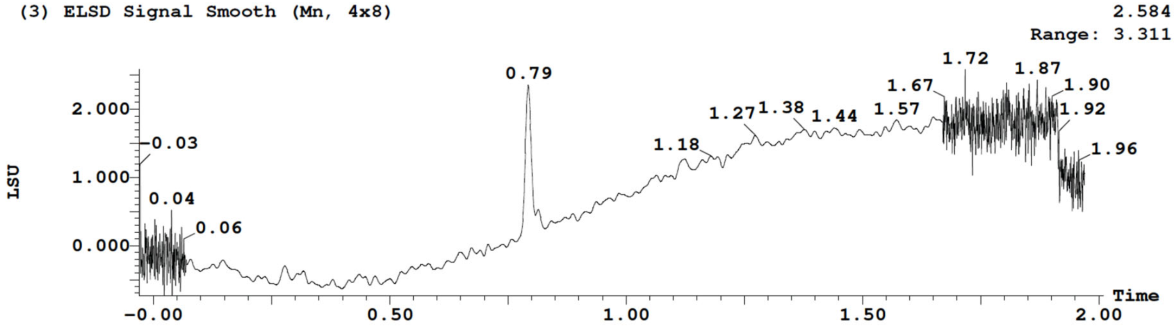

Peak Number	Compound	Time	AreaAbs	Area %Total	Width	Height	Mass Found

Peak ID	Compound	Time	Mass Found
17	Found	1.22	205

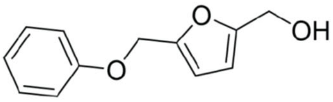

10

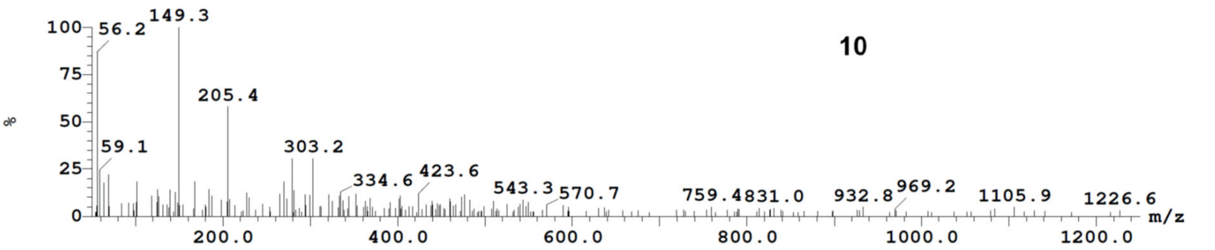

Figure S15. LC–MS analysis of (5-(phenoxymethyl)furan-2-yl)methanol (10). The UPLC chromatogram confirms a single major component (~97% purity). In the ESI–MS, the predominant ion is observed at m/z 187, corresponding to a dehydrated protonated molecule [M+H–H_2O^+ (loss of H_2O from the $[M+H]^+$ of M_w 204).

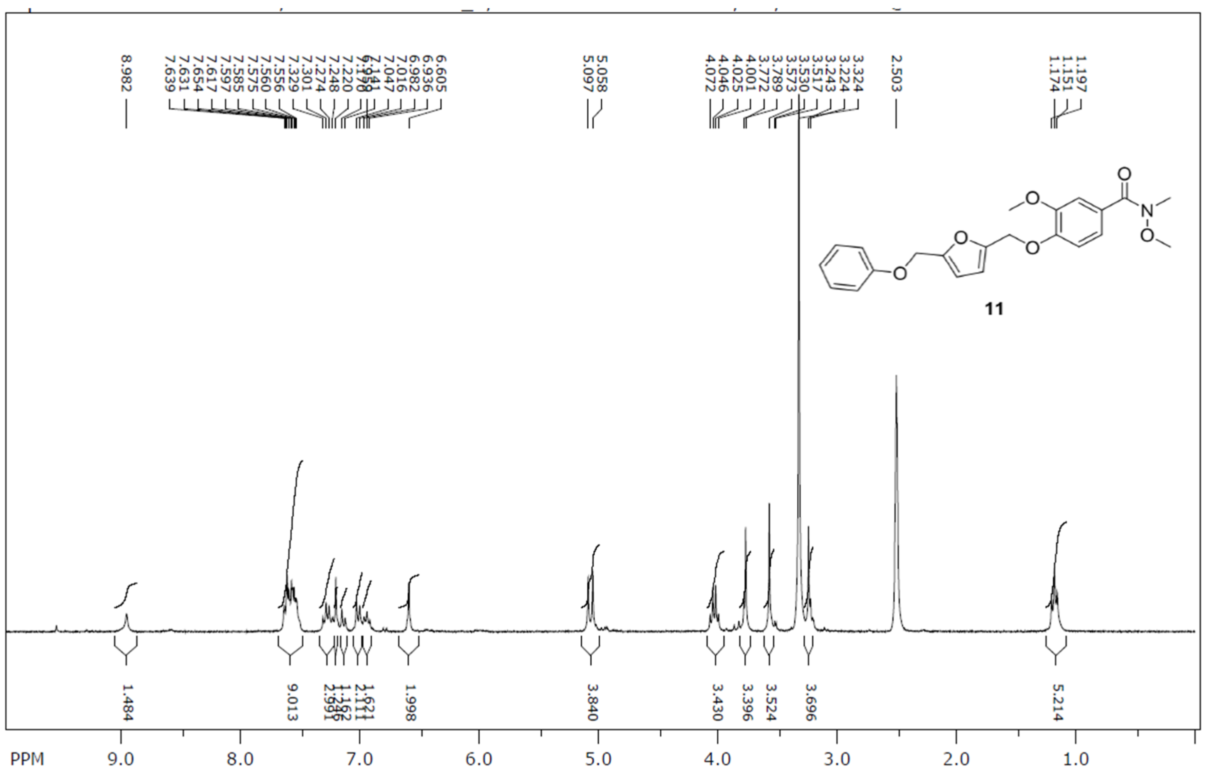

Figure S16. ^{1}H NMR spectrum of N,3-dimethoxy-N-methyl-4-((5-(phenoxymethyl)furan-2-yl)methoxy)benzamide (11) in DMSO-d6 at 300 MHz. Three singlets at δ 3.78, 3.57, and 3.23 integrate to 3H each and correspond to the 3-methoxy (aromatic OCH_3), N-methoxy, and N-methyl groups of the Weinreb amide, respectively. The aromatic protons of the benzamide ring and the phenoxy-furan moiety appear in the δ 6.6–7.3 region, and the benzylic O–CH_2– linkage protons resonate around δ 5.08 (overlapping signals due to the phenoxymethyl and aryl–OCH_2–furan methylenes).

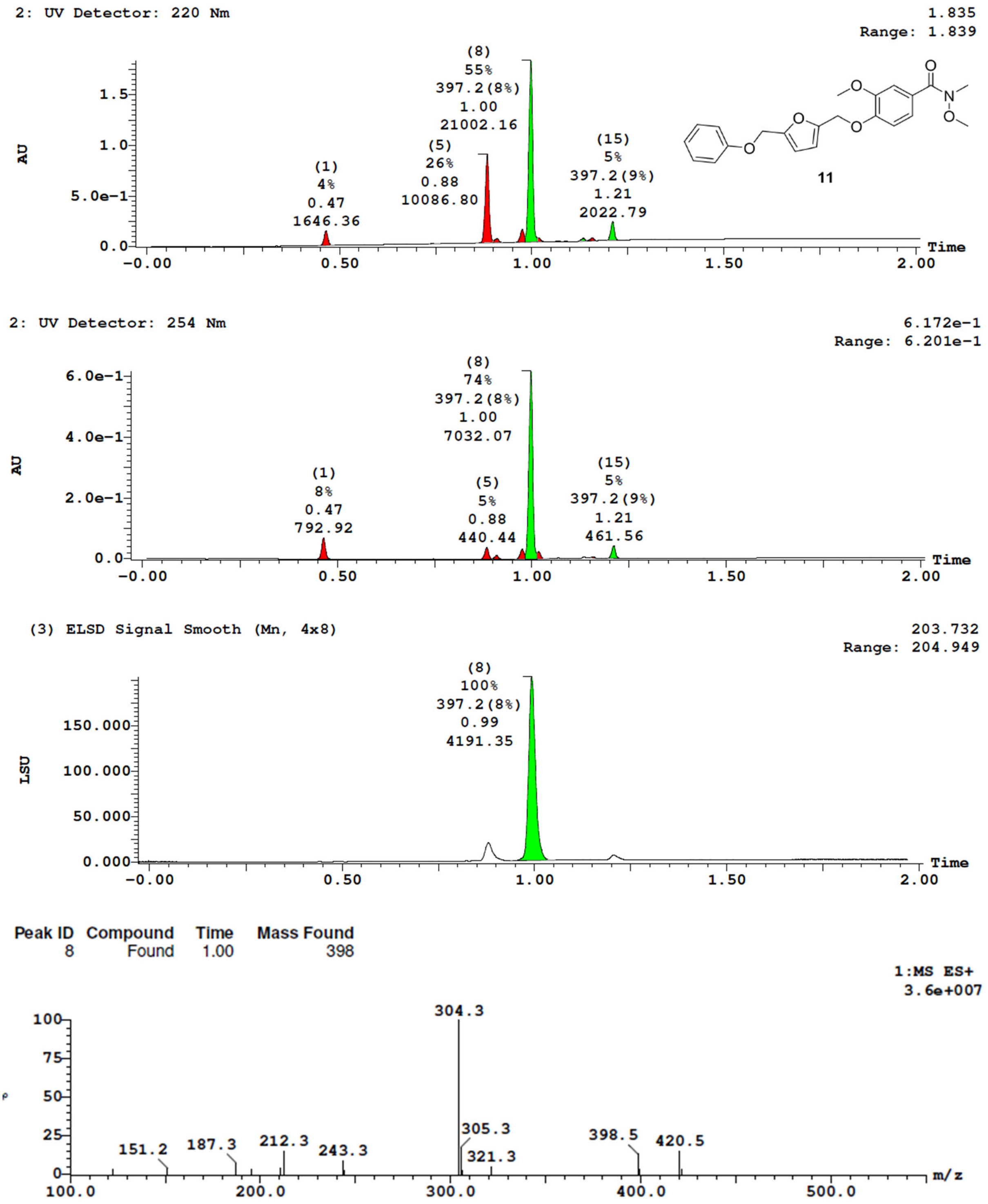

Figure S17. LC–MS trace of N,3-dimethoxy-N-methyl-4-((5-(phenoxymethyl)furan-2-yl)methoxy)benzamide (11). The protonated molecular ion is observed at m/z 398 ($[M+H]^+$). The LC chromatogram reveals that the major product peak constitutes only ~55% of the total area (220 nm), indicating the presence of a significant impurity (consistent with residual triphenylphosphine oxide from the reaction).

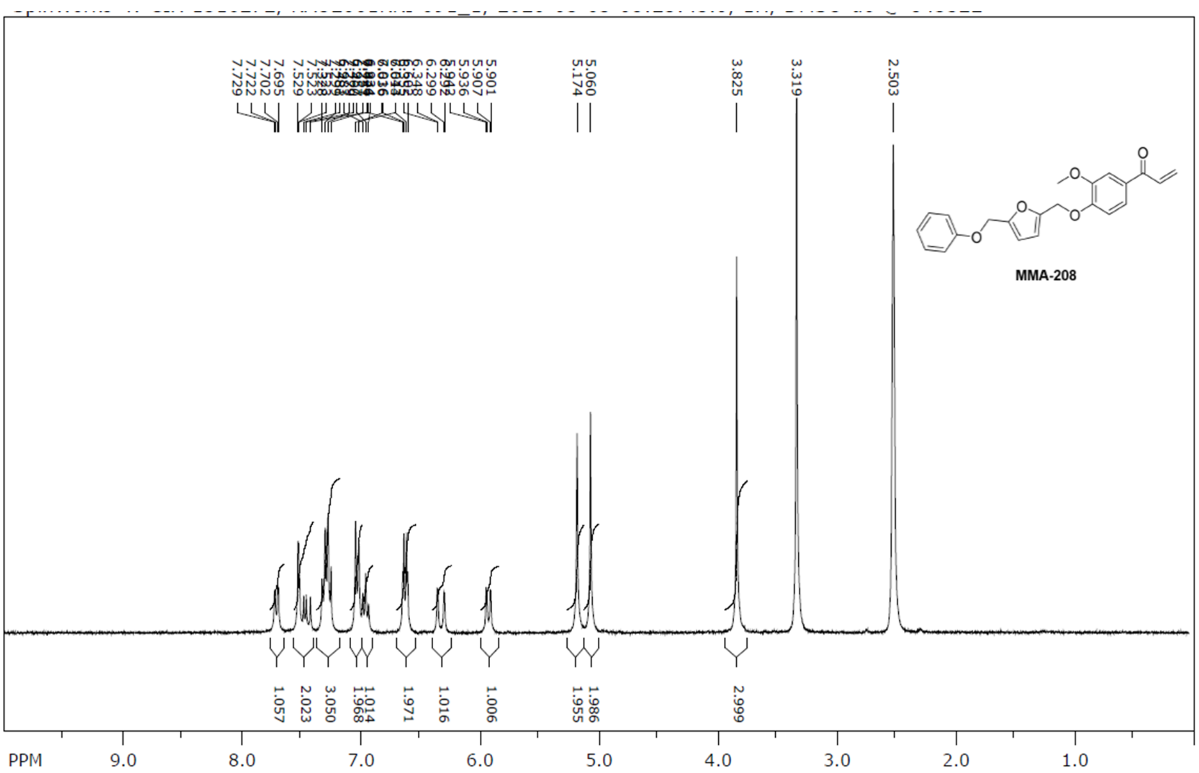

Figure S18. ^{1}H NMR spectrum of 1-(3-methoxy-4-((5-(phenoxymethyl)furan-2-yl)methoxy)phenyl)prop-2-en-1-one (MMA-208) in DMSO-d_6, 300 MHz. Two distinct benzyloxymethylene groups are observed as singlets at δ 5.17 and 5.06 (2H each), corresponding to the Ar–O–CH_2–furan linkage and the phenoxymethyl–CH_2–furan substituent, respectively. The aromatic and vinylic protons appear in the δ 6.32–7.71 range, and the aromatic OCH_3 group gives a singlet at δ 3.83.

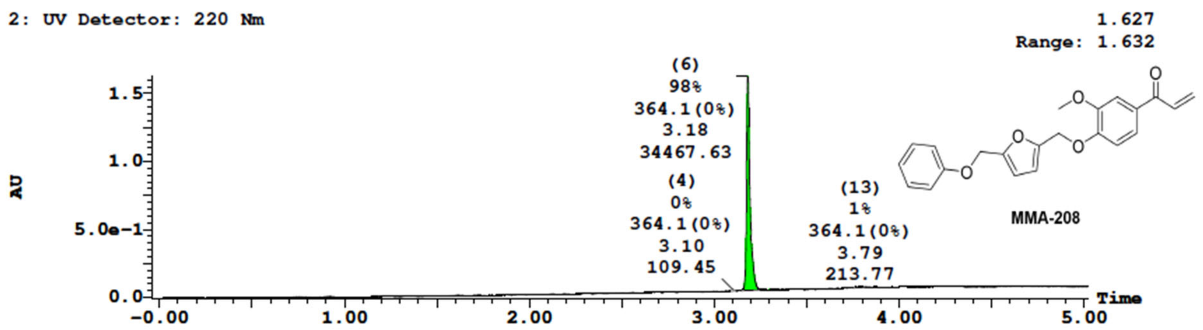

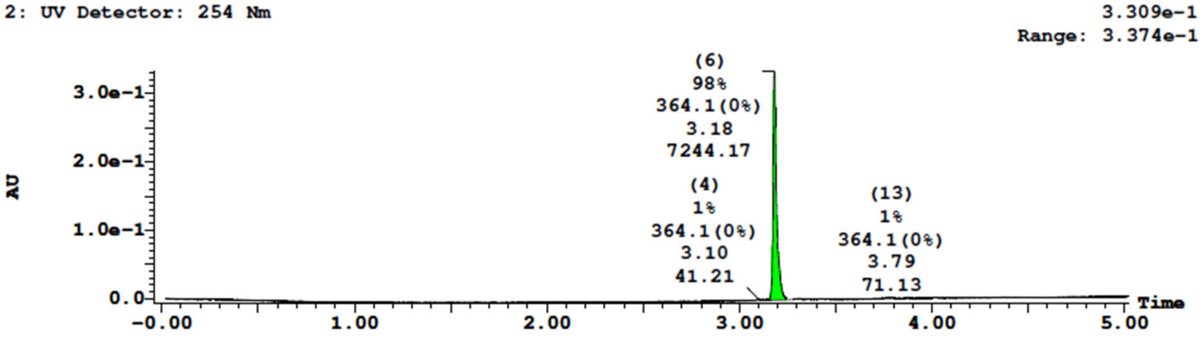

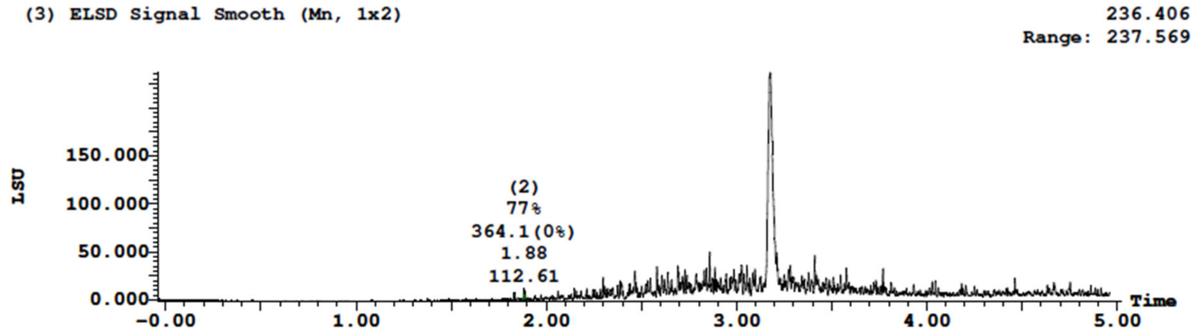

Peak ID	Compound	Time	Area %BP	Mass Found
6	Found	3.18	100	Not Found

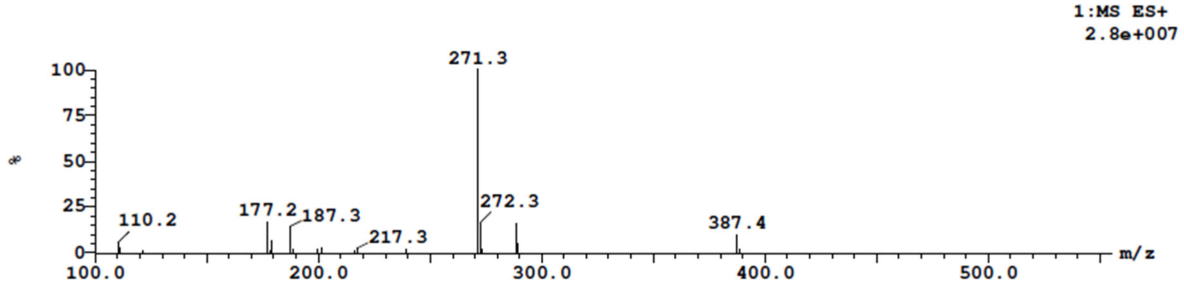

Figure S19. LC–MS analysis of 1-(3-methoxy-4-((5-(phenoxymethyl)furan-2-yl)methoxy)phenyl)prop-2-en-1-one (MMA-208). The UPLC–UV chromatogram (220 nm) shows a single principal peak (~98% purity). The positive-ion mass spectrum exhibits an ion at m/z 387, consistent with the sodium adduct of the molecule ($[M+Na]^+$).

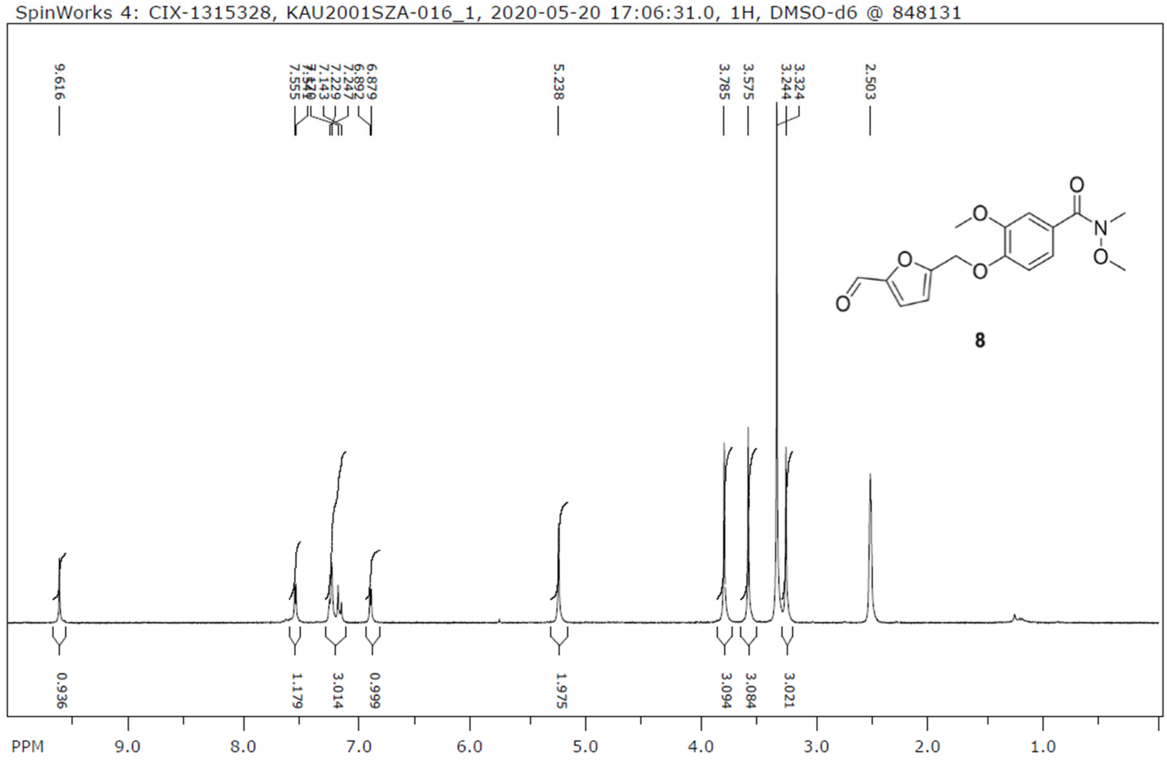

Figure S20. ^{1}H NMR spectrum of 4-((5-formylfuran-2-yl)methoxy)-N,3-dimethoxy-N-methylbenzamide (8) in DMSO-d_6 at 300 MHz. The formyl proton on the furan ring appears as a singlet at δ 9.62. The spectrum also displays the expected three methoxy/methyl singlets for the N,O-dimethylamide functionality (δ 3.79 for aromatic OCH_3, 3.57 for N–OCH_3, and 3.24 for N–CH_3), along with the benzylic O–CH_2– singlet at δ 5.24 and aromatic/furan proton signals in the δ 6.89–7.55 range.

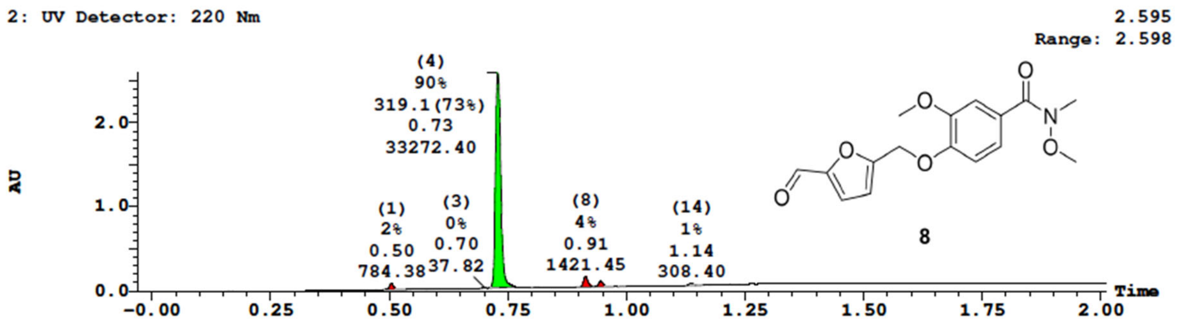

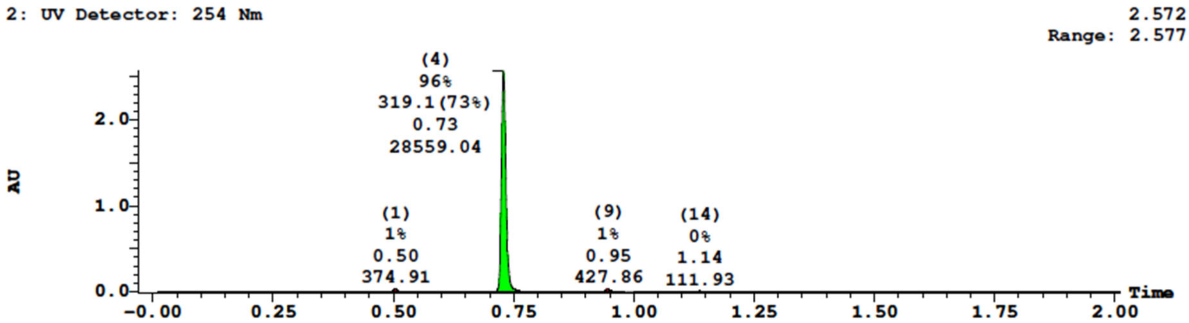

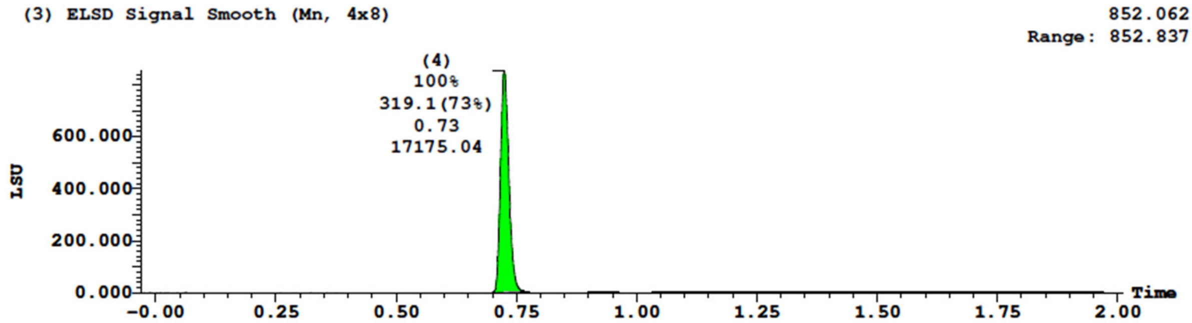

Peak Number	Compound	Time	AreaAbs	Area %Total	Width	Height	Mass Found
4	Found	0.73	2e+004	100.00	0	9e+005	319.1056

Peak ID	Compound	Time	Mass Found
4	Found	0.73	320

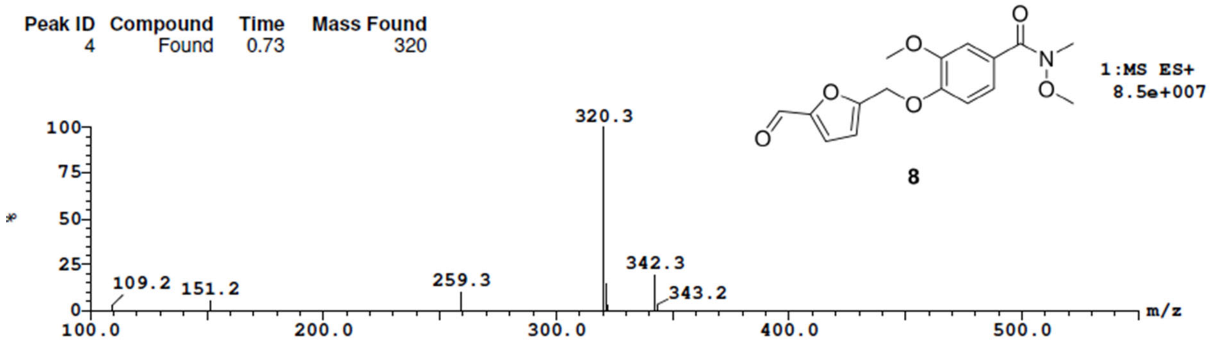

Figure S21. LC–MS analysis of 4-((5-formylfuran-2-yl)methoxy)-N,3-dimethoxy-N-methylbenzamide (8). The ESI mass spectrum shows the $[M+H]^+$ ion at m/z 320, and the LC chromatogram displays a single major peak (~96% area purity) corresponding to the desired benzaldehyde product.

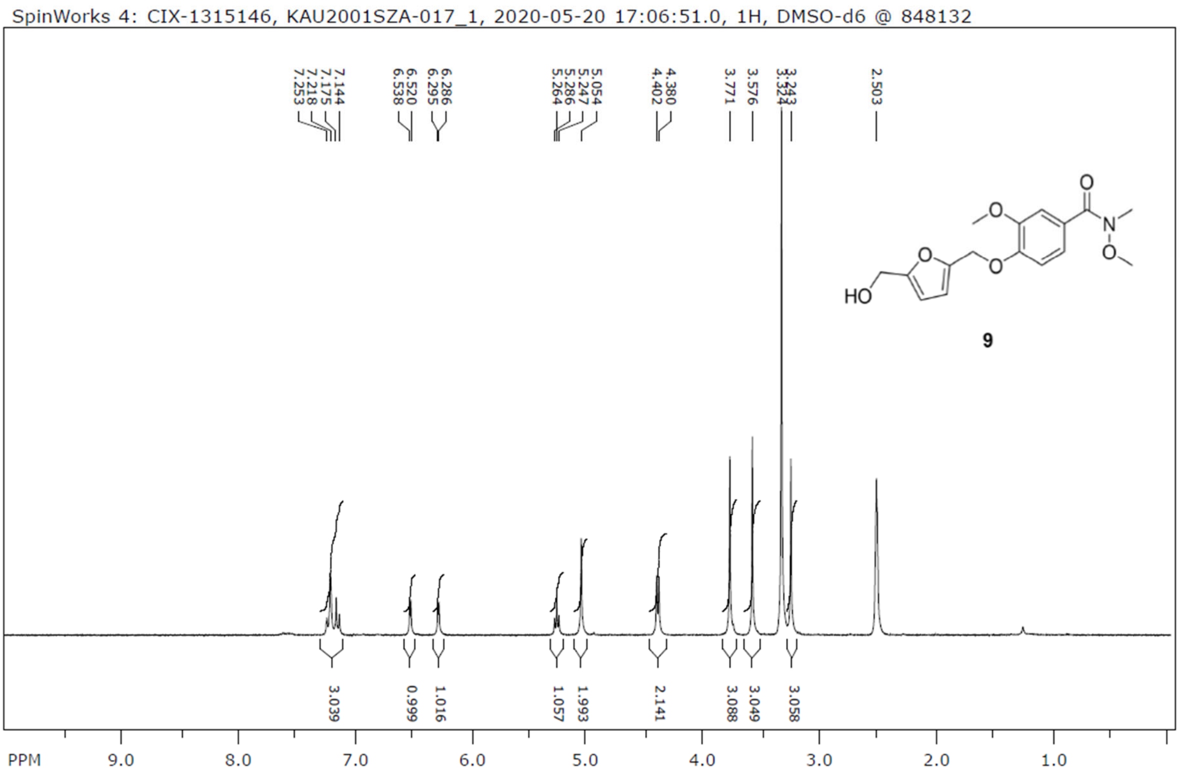

Figure S22. ^{1}H NMR spectrum of 4-((5-(hydroxymethyl)furan-2-yl)methoxy)-N,3-dimethoxy-N-methylbenzamide (9) in DMSO-d_6, 300 MHz. The furan–CH_2OH moiety is evident from a doublet at δ 4.39 (2H, CH2, J ≈ 5.7 Hz) and an exchange-broadened triplet at δ 5.27 (1H, OH, J ≈ 5.8 Hz). The three methoxy/methyl singlets of the N,3-dimethoxy-N-methylbenzamide group are observed at δ 3.77, 3.58, and 3.24, and the remaining aromatic and furan protons appear at δ 6.29–7.29.

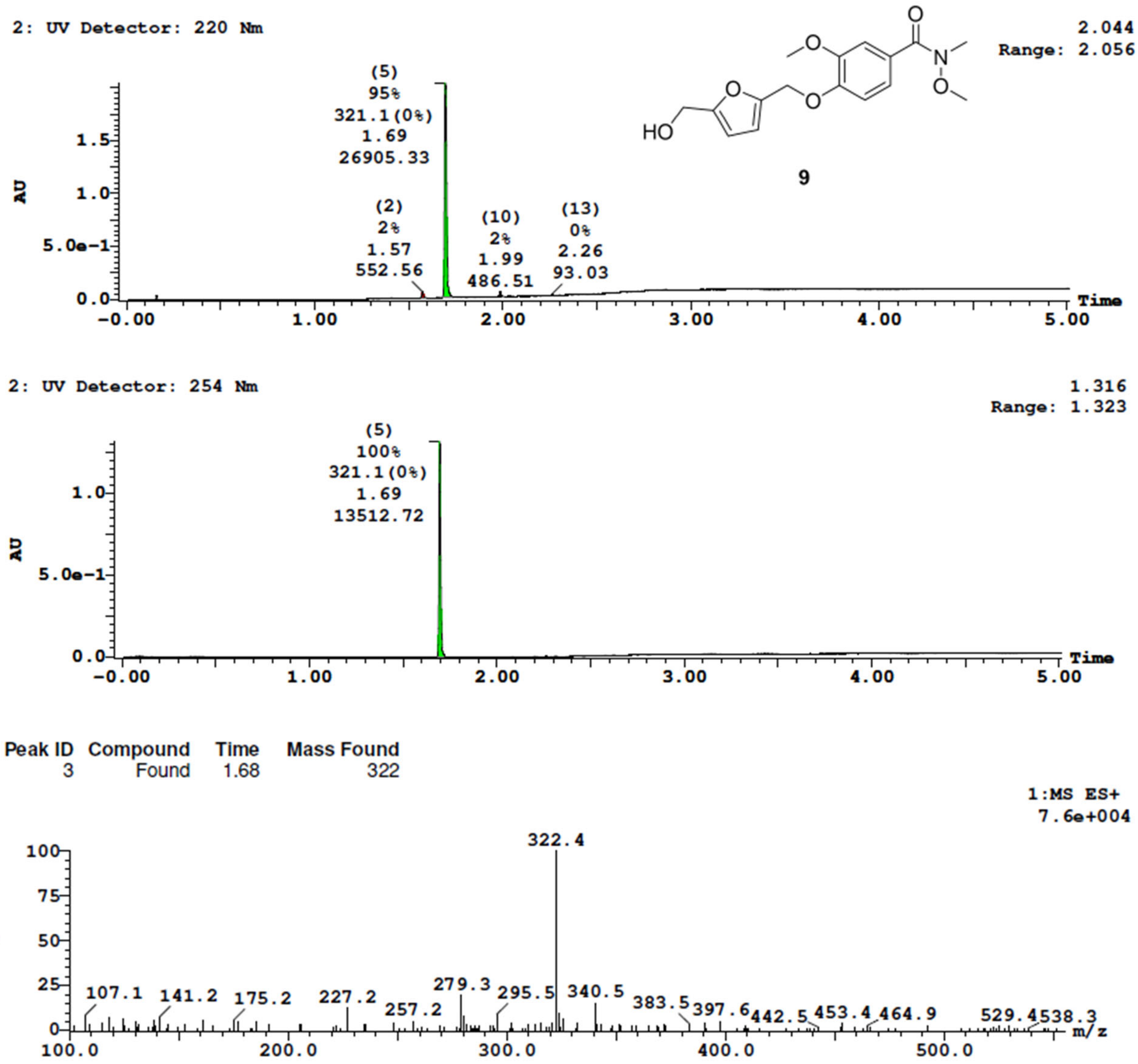

Figure S23. LC–MS of 4-((5-(hydroxymethyl)furan-2-yl)methoxy)-N,3-dimethoxy-N-methylbenzamide (9). The positive ESI–MS shows a prominent sodium adduct at m/z 344 ($[M+Na]^+$). The UPLC chromatogram (220 nm) reveals a predominant product peak (~95% purity) with only minor impurities.

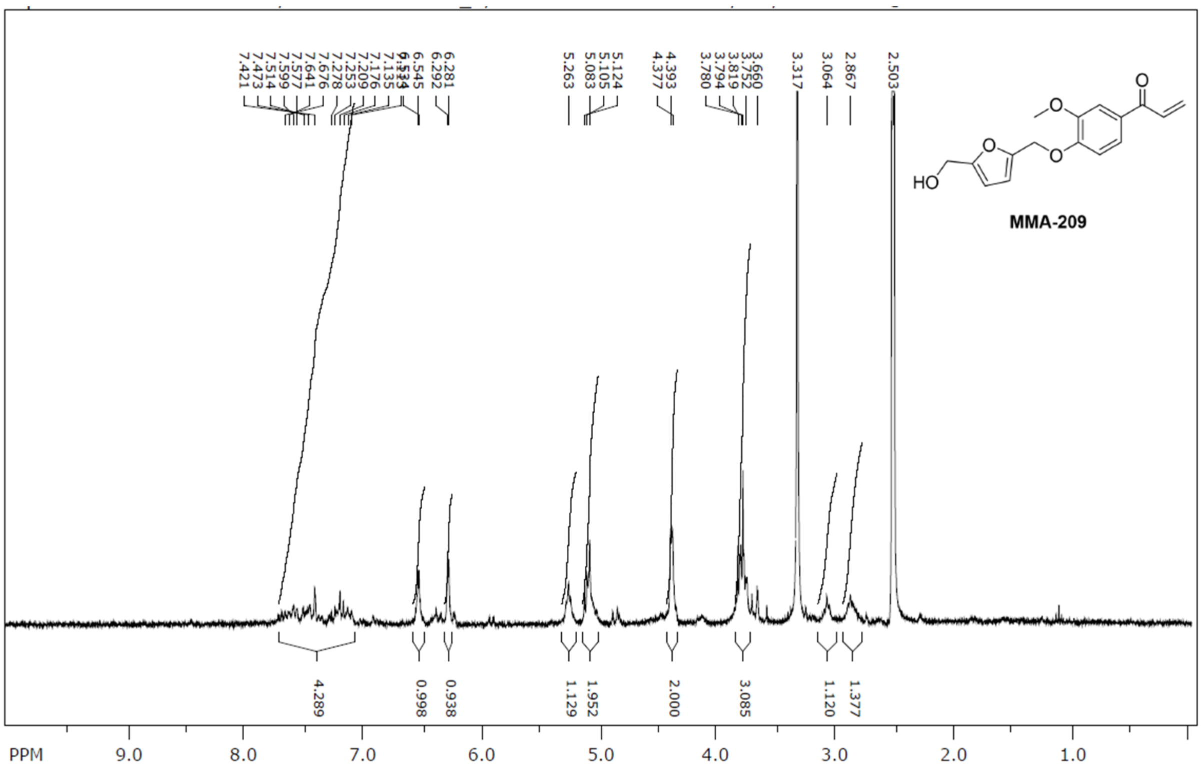

Figure S24. ^{1}H NMR spectrum of 1-(4-((5-(hydroxymethyl)furan-2-yl)methoxy)-3-methoxyphenyl)prop-2-en-1-one (MMA-209) in DMSO-d_6 at 300 MHz. The aromatic and olefinic protons integrate to four protons between δ 7.05–7.77. The furan-CH_2–O–aryl linkage and the furan CH_2OH protons give rise to signals in the δ 4.4–5.2 region (with the –OH proton likely overlapping in this range). The 3-methoxy group on the aromatic ring appears as a singlet at δ ~3.8.

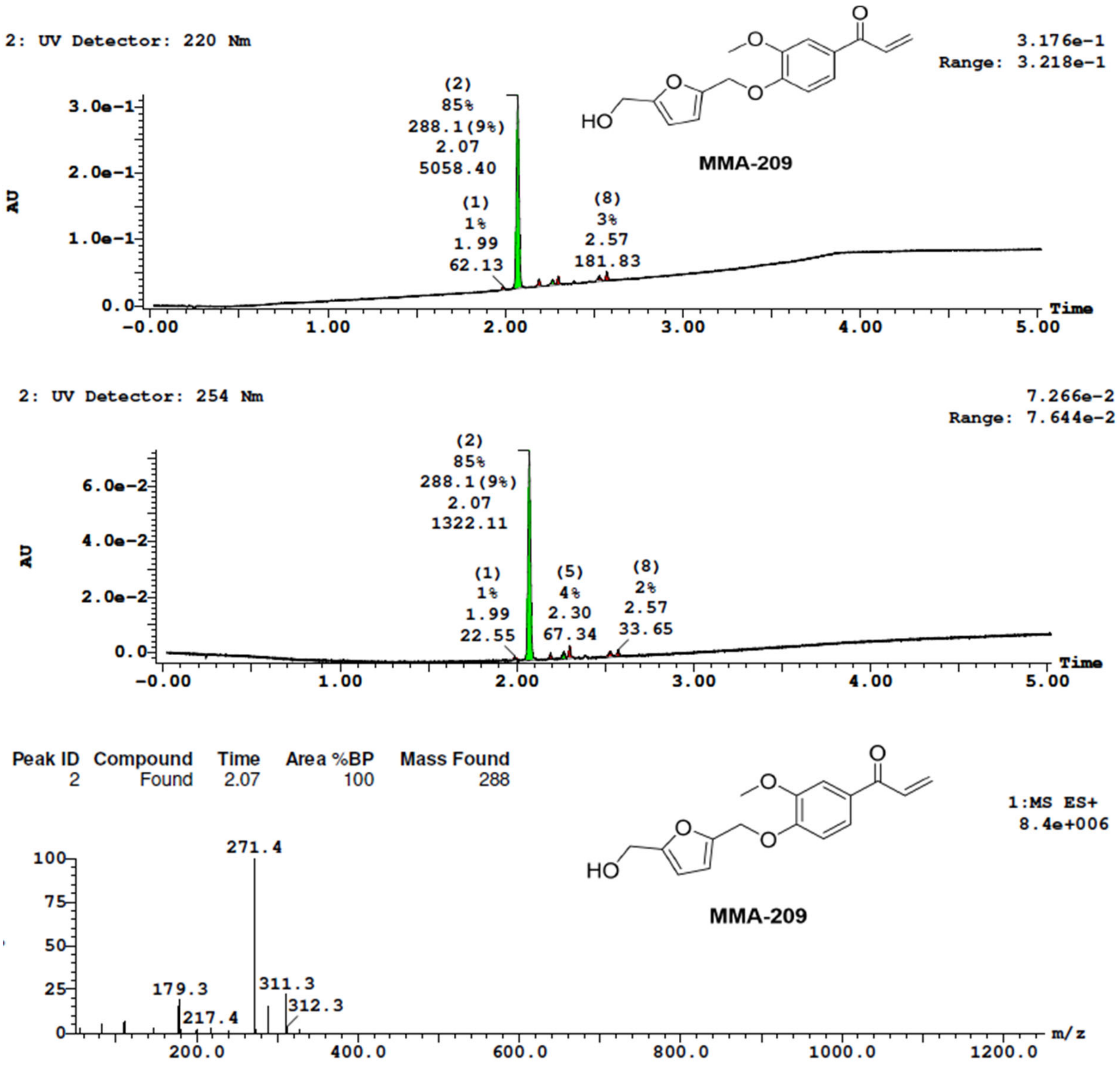

Figure S25. LC–MS analysis of 1-(4-((5-(hydroxymethyl)furan-2-yl)methoxy)-3-methoxyphenyl)prop-2-en-1-one (MMA-209). The product exhibits an ion at m/z 311, corresponding to the sodium adduct ($[M+Na]^+$) in the positive-ion mass spectrum. The LC chromatogram indicates a predominant product peak with approximately 85% purity, alongside minor impurity peaks.
